# Supplementary material for: Obstructive Sleep Apnea and Nonalcoholic Fatty Liver Disease in the General Population: A Cross-Sectional Study Using Nationally Representative Data
Source: Int J Environ Res Public Health. 2022 Jul 9;19(14):8398. doi: 10.3390/ijerph19148398 (PMC9321646; doi:10.3390/ijerph19148398)
Supplement: Supplementary file 1 [file ijerph-19-08398-s001.zip › ijerph-1783233-supplementary.pdf]

Supplementary Table S1. Multivariable-adjusted OR (95% CI) for NAFLD by the risk of having OSA according to the usage of the cholesterol-lowering drug and the presence of diabetes.

	Low	Intermediate	High	*p*
**By usage of cholesterol-lowering drug**				
**All**				
No (n = 251)	1	2.83 (0.94–8.47)	5.79 (1.04–32.34)	0.027
Yes (n = 796)	1	0.88 (0.53–1.45)	0.90 (0.35–2.27)	0.773
**BMI ≥ 25kg/m²**				
No (n = 99)	1	7.89 (1.84–33.99)	38.32 (3.54–415.04)	0.001
Yes (n = 387)	1	0.91 (0.49–1.69)	0.80 (0.31–2.07)	0.646
**BMI < 25kg/m²**				
No (n = 152)	1	0.32 (0.03–3.20)	1.07 (0.06–18.02)	0.806
Yes (n = 409)	1	0.70 (0.28–1.79)	1.85 (0.34–10.11)	0.719
**By presence of diabetes**				
	Low	Intermediate	High	*p*
**All**				
No (n = 3,547)	1	1.87 (1.34–2.62)	2.83 (1.49–5.38)	<0.001
Yes (n = 728)	1	0.85 (0.48–1.49)	1.87 (0.71–4.95)	0.333
**BMI ≥ 25kg/m²**				
No (n = 1,191)	1	2.03 (1.44–2.85)	2.99 (1.50–5.95)	<0.001
Yes (n = 400)	1	0.99 (0.46–2.12)	1.91 (0.61–5.98)	0.242
**BMI < 25kg/m²**				
No (n = 2,356)	1	1.51 (0.71–3.24)	3.10 (0.87–11.09)	0.085
Yes (n = 328)	1	0.79 (0.35–1.75)	4.40 (1.01–19.22)	0.41

Multivariate model considered age, sex, residence, education, household income, smoking, physical activity, and body mass index as covariates. The risk of having OSA was measured using STOP-Bang questionaries. OR = odds ratio, CI = confidence interval, OSA = obstructive sleep apnea, NAFLD = non-alcoholic fatty liver disease, STOP-Bang = snoring, tiredness, observed apnea, high BP, BMI, age, neck circumference, and male gender.
